# GEL-MAN Hydrogel Loaded With Triamcinolone Acetonide for the Treatment of Osteoarthritis

**DOI:** 10.3389/fbioe.2020.00872

**Published:** 2020-07-31

**Authors:** Kai Chen, Shanzhu Li, Feng Yuan, Pengfei Sun, Yingying Zhang

**Affiliations:** ^1^Department of Orthopedic Surgery, Tongji Hospital, Tongji University School of Medicine, Shanghai, China; ^2^Key Laboratory for Organic Electronics and Information Displays & Institute of Advanced Materials, Nanjing University of Posts and Telecommunications, Nanjing, China; ^3^Department of Nephrology, Tongji Hospital, Tongji University School of Medicine, Shanghai, China

**Keywords:** osteoarthritis, triamcinolone acetonide, GEL-MAN hydrogel, treatment, hydrogen peroxide (H_2_O_2_)

## Abstract

One of the major challenges for the treatment of osteoarthritis (OA) with therapeutic drugs is the short half-life of drugs in the joint cavity. The severity of OA often fluctuates with time and inflammatory factors. Here, we describe the use of a hydrogel material, named Gel-Man, to solve the problem of rapid release of drugs. Gel-Man could encapsulate a series of therapeutic drugs and be degraded by hydrogen peroxide. It could be decomposed, and release drugs controlled by the concentration of hydrogen peroxide in the arthritic joint cavity. This hydrogel loaded with triamcinolone acetonide (TA) could slowly release the drug upon exposure to hydrogen peroxide in the joint cavity in patients suffering from osteoarthritis. The combination of TA and GEL-MAN hydrogels can slowdown the progression of degenerative change of osteoarthritis by maximizing the therapeutic efficacy and prolong the duration of drug treatment.

## Introduction

Osteoarthritis is the most common form of arthritis affecting approximately 237 million people (March et al., 2014; Disease et al., 2016). It is a chronic orthopedic disease caused by hyperostosis and articular cartilage degeneration. Clinical manifestations include knee join pain, frictional sounds during movement, limited joint activity, joint swelling and joint deformity et al. Some patients even have symptoms such as lameness and knee varus deformity, that seriously affect their health and quality of life.

There are various risk factors for OA, such as age, obesity, gender, and heredity (Cross et al., 2014). Abnormal load on joints may be associated with osteoarthritis (Stubbs et al., 2016; Thomas et al., 2017). It is currently believed that the occurrence and development of OA are associated with inflammation. Studies have shown that cytokines that involved in the pathophysiology of OA include TNF, IL-1β, IL-6, IL-15, IL-17, IL-18, IL-21, and LIF (Kapoor et al., 2011). The specific pathogenesis of osteoarthritis is not clear, but it might involve NF-κB, p38 MAPK, and Wnt/β-catenin cell signaling pathways, macrophage autophagy clearance, multiple transcription factors, and miRNAs. The imbalance between pro-inflammatory and anti-inflammatory factors, estrogen, mechanical stress and other extracellular factors may also involve in the pathogenesis of OA (Kato et al., 2014). At present, a variety of treatments are available. The most common one is the intra-articular injection of drugs, such as sodium hyaluronate, medical ozone, chondroitin sulfate, and proprietary Chinese medicine. The advantage of intra-articular injection is that, drugs directly reach the affected joints and high concentration of drugs in the joint cavity, which lowers the systemic side effects. In addition, joint fluid can be extracted for examination during joint cavity injection. The extraction of the joint fluid can not only relieve the pressure of the joint but also alleviate the symptoms of arthritis.

Matrix genes, such as aggrecan, collagen type II, as well as key pathological proteases, such as collagenases (MMP1, MMP13) and aggrecanases (ADAMTS4, ADAMTS5) are important factors for alteration of articular cartilage (Scanzello and Goldring, 2012). Matrix metalloproteinases (MMPs) and aggrecanases (ADAMTS4, ADAMTS5) are enzymes that involved in the turnover and degradation of articular matrix. They are the risk factors of OA (van den Berg, 2011). Collagen II is an important fibrillar protein in cartilage that affects the structure of functional skeleton (Arslan et al., 2018). The increase of MMPs and ADAMTS5 will cause the degradation of concentrations of fragmented type II collagen, AGGRECAN in articular cartilage. As a result, collagen will be degraded and proteoglycan content reduced when OA occurre.

Polyvinyl alcohol (PVA) could be used in a certain ratio with drugs to synthesize a hydrogel which can release therapeutic drugs at the site of cartilage injury and promote joint recovery. Hydrogel can also be manipulated into a certain shape as needed and naturally integrated with the surrounding tissues. By adjusting the ratio of polyvinyl alcohol, the hydrogel can be synchronized for degradation and regeneration of cartilage tissue with strong controllability. When the PVA ratio is increased, the degradation process of cartilage tissue is slowed down, thereby prolong the regeneration time of chondrocytes.

Hydrogel is a type of semi-solid carbohydrate-based material with 3D networks. Due to the properties of tunable physiochemistry, it has been known as one of the most attractive *in situ* drug delivery systems (Sun et al., 2017). Non-covalent interaction is essential for the structure of carbohydrate-based materials. Based on our previous studies, the isomerism effect plays a great role for interactions between biological molecules, such as glycopolymers. Benzoxaborole (BOB) is a Lewis acid (pKa = 7.3), which could bind to carbohydrates to form dynamic covalent bonds via oxacarbocyclic skeleton with a cis-diol-hydroxyl handle (Lin et al., 2014). P-1-Man (C1-position hydroxyl group) is a sugar isomer, the glycopolymer carrys it as a pendant group. GEL-MAN is a hydrogel formed by BOB-containing polymers (BOB-P-1-Man). This hydrogel material can encapsulate a variety of therapeutic drugs and be degraded by hydrogen peroxide. Therefore, this hydrogel material can be decomposed by hydrogen peroxide in the joint cavity to slowly release the encapsulated drugs for the treatment of arthritis. In this study, we used the corticosteroid Triamcinolone acetonide (TA) as a model drug and loaded it in the hydrogel. We demonstrated that TA is released in response to the concentration of hydrogen peroxide in the synovial fluid in the arthritic cavity using a rat model of OA. We also demonstrated that the breakdown of hydrogels and the release of encapsulated drugs are associated with the severity of osteoarthritis. Our study provides a rationale for application of the hydrogel as a potential therapeutic drug delivery system for OA treatment.

## Materials and Methods

### Gel-Man Preparation and Rheological Behavior

1.0 mL polyaddition of (2-oxooxazolidin-5-ylmethoxy) benzene (PBOB) (100 mg/mL in water, pH = 8.0) was mixed with 1.0 mL P-1-Man (mannoses’ anomeric position) (100 mg/mL in phosphate buffer saline (PBS), pH = 7.4, salt concentration:100 mM) at room temperature. The storage and loss modulus were measured with a plate-to-plate rheometer (MCR 302, Anton Paar, Ashland, VA, United States) using a 25 mm plate under a constant strain of 1% and frequency of 10 rad/s. 1.0 mL of premixed TA (1.43 mg/ml) was added to 1.0 mL of PBOB solution. GEL-TA was rapidly formed at room temperature.

### Animals and OA Mouse Model

Male SD rats, 8 weeks old, purchased from Shanghai SLAC Laboratory Animal Co., Ltd. (Shanghai, China), were housed in a constant humidity (50% ± 5%) and temperature (25°C ± 2°C) environment on a 12 h light/dark cycle. All protocols and studies were approved by the Animal Ethics Committee of our institution and carried out in accordance with the guide for Institutional Animal Care and Use Committee and National Institutes of Health guide for the care and use of laboratory animal. Rats were randomly divided into five groups. (i) control group; (ii) Model group; (iii) GEL-MAN hydrogel group; (iv) TA group; and (v) TA + GEL-MAN hydrogel group. Animals were observed daily for activity and weighed once a week.

Before experiment, all the rats were uniformly fed in the same living environment for 1 week. After confirming that the experimental rats had no significant difference in general conditions such as food intake, activity level, and mental status, OA model was established by the classical surgical modeling method of Hulth method. Briefly, after intraperitoneal injection of sodium pentobarbital for anesthesia, rats were fixed in supine position on the workbench. The hair around the knee joints of both hind limbs was cut off, the skin was exposed and thoroughly disinfected using iodophor. Then, the medial longitudinal approach of the knees of the hind limbs of the rats was taken. The anterior and posterior cruciate ligaments of the knee joint and the medial collateral ligament were surgically removed, and the medial meniscus of the knee joint was removed. The operation of the knee joint was avoided to avoid damage to the knee cartilage. After the successful operation, the rats were intramuscularly injected with 80,000 U of penicillin sodium once a day, continued for 3 days after surgery to prevent postoperative infection. After 72 h of surgery, the rats were forced to perform activities once a day, at least 30 min each time, for 6 weeks.

### Drug Injection

After 72 h of operation, animals were given weekly injections of 70 μl of the solutions via a 27-gauge needle in their OA-induced knee joint for 3 weeks.

### Histology and Immunohistochemical Staining

The joint samples, heart, liver, spleen, lung, kidney and skin tissue were collected from the rats and routine HE staining was performed. Knee joint samples were fixed in 4% (v/v) neutral buffered formalin for 24 h and decalcified for 1 month at room temperature in neutral 10% EDTA solution. Subsequently, the sample was dehydrated in an ethanol gradient, clarified, and embedded in a paraffin block. Tissue sections (8 μm) were prepared. Six representative sections of the joints from various depths were mounted on glass slides, stained with Safranin-O, and photographed under a microscope (BH2 UMA, Olympus). After overnight incubation with MMP13 (ABCAM, ab39012), COL2A1 (ABCAM, ab34712), ADAMTS-5 (ABCAM, ab41037), and AGGRECAN (ABCAM, ab3778) primary antibodies at 4°C, sections were incubated with secondary antibodies for 2 h at room temperature. Color development was performed using a DAB substrate system. Hematoxylin was used to stain the nucleus of cells.

### Scoring of OA

Semi-quantitative histopathological grading was performed using a revised Chambers scoring system established by the OARSI (Osteoarthritis Research Society International) histopathology initiative as a standard method for grading rodent cartilage degeneration. Based on this system, paraffin sections from each sample were scored after safranin-O staining. Histological grading was performed in 4 areas: medial femoral condyle, medial tibial plateau, lateral femoral condyle, and lateral tibial plateau. The samples were independently evaluated by trained researchers blinded to the histological data. In this analysis system, the score is a combination of grade and stage from 0 to 24, the depth of damaging progression of OA’s cartilage is range from 0 to 6 (grade). The horizontal damaging extent of cartilage is defined from 0 to 4 (stage).

### Statistical Analysis

All experiments were repeated at least 3 times and data were expressed as mean ± SEM. One-way analysis of variance (ANOVA) was performed to analyze the significance between different sets of data with GraphPad Prism 5.0. *p* value of ≤0.05 was defined statistically significant.

## Results

### Basic Properties of GEL-MAN Hydrogel

**FIGURE 1:**
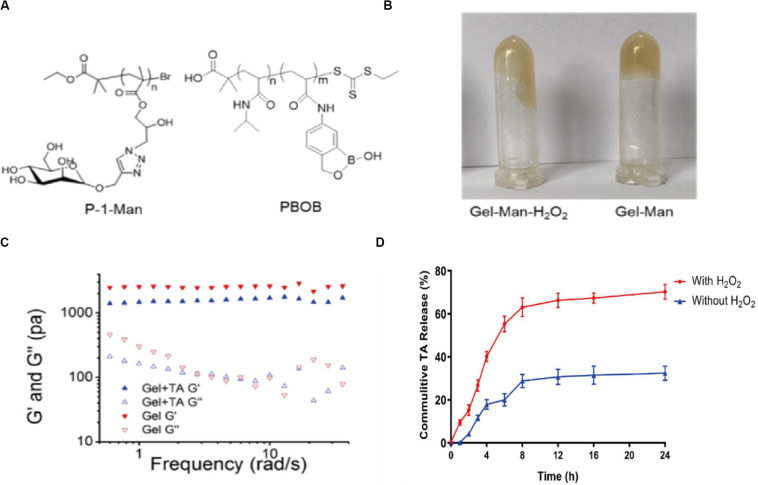
Basic properties of GEL-MAN hydrogel. **(A)** Chemical structure of GEL-MAN: **(B)** GEL-MAN with H_2_O_2_ **(C)** Frequency-dependent storage moduli (G’) and loss moduli (G”) of GEL-MAN and GEL-MAN with TA: **(D)** *In vitro* release kinetics of TA in response to H_2_O_2_. Data are means ± SEM (*n* = 3).

GEL-MAN (Figure 1A) with 10% total weight content were prepared according to previously reported procedures (Mingchang et al., 2014; Sun et al., 2018). Briefly, The hydrophobic drug can be encapsulated in the hydrophobic core of the hydrogel during formation of the hydrogel. A drug-loaded hydrogel can be administered for injection into the joint cavity. In the joint, high content of hydrogen peroxide in arthritic join cavity can break down the hydrogel, resulting in a rapid drug release response (Figure 1B). We encapsulated TA in GEL-MAN hydrogel (10% w/v). Through literature research and preliminary experiments, we chose to use a hydrogel prepared with 20 mg/ml TA for *in vitro* and *in vivo* studies. Rheological behavior of the GEL-MAN hydrogel, in the range of 0.2 to 10 rad/s, the storage (G’) moduli of GEL-MAN were larger than those of the loss (G”) moduli, indicating that GEL-MAN exhibits a characteristic of a solid material (Figure 1C). Interestingly, the storage modulus G’ of GEL-MAN reaches 1180 Pa. In the whole frequency region, G’ was higher than loss modulus G” for both GEL-MAN and GEL-MAN with TA as solids. In short, our hydrogelation experiment indicates that the binding ability of GEL-MAN with TA was strong. Then we studied GEL-MAN under simulated inflammatory conditions. The effect of the release of TA from the gel. The drug-loaded hydrogel was incubated in PBS with or without adding an appropriate amount of hydrogen peroxide to simulate the joint cavity environment. It is worth noted that the release of TA increased under hydrogen peroxide conditions compared to PBS (pH 7.4) (Figure 1D).

### Toxicity of GEL-MAN Hydrogel *in vivo*

**FIGURE 2:**
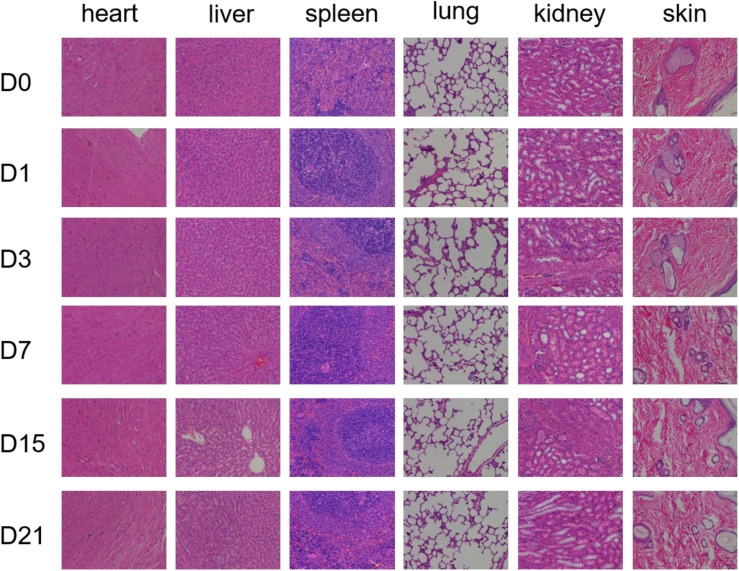
Pathological observation of toxicity of GEL-MAN hydrogel *in vivo*. The HE staining of different tissues were performed on day 0, 1, 3, 7, 15, and 21.

The safety of the material *in vivo* is critical. We investigated the *in vivo* biocompatibility of GEL-MAN by injecting hydrogel into the join cavity of SD rats. The results show that the GEL-MAN does not cause inflammatory reactions. GEL-MAN hydrogel has no observable toxicity to heart, liver, spleen, lung, kidney, and skin tissues (Figure 2). This result indicates that GEL-MAN hydrogel is safe and feasible to use as potential tissue engineering material.

### TA Loaded With GEL-MAN Hydrogel for Treatment of Osteoarthritis

**FIGURE 3:**
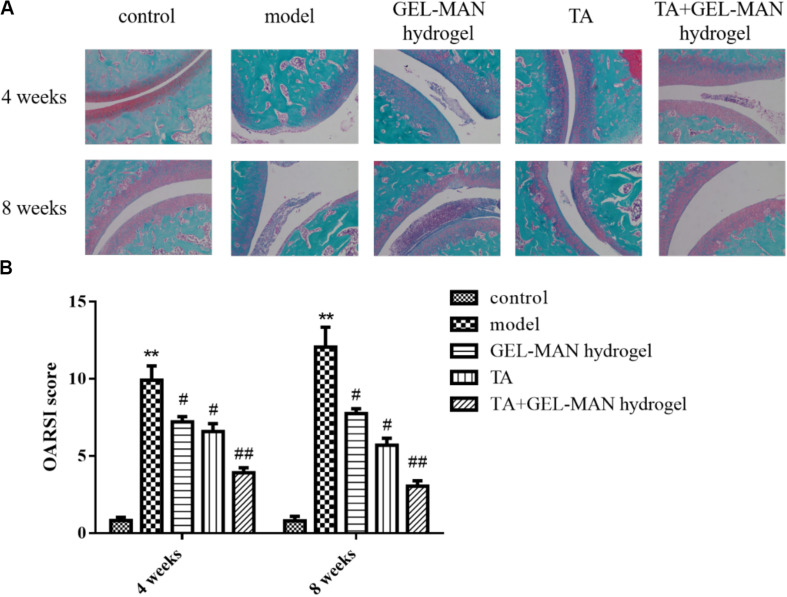
The evaluation of TA loaded with GEL-MAN hydrogel for the treatment of OA at 4 and 7 weeks via Safranin-O staining and OARSI. **(A)** Safranin-O staining on cartilage samples at 4 and 8 weeks after OA induction: **(B)** OARSI scores of cartilage samples at 4 and 8 weeks after OA induction. Data are means ± SEM (*n* = 6). # indicates *p* < 0.05 compared with the model group, ## indicates *p* < 0.01 compared with the model group, ** indicates *p* < 0.01 compared with the control group.

The hydrogel-injected rats showed a significant decrease in the OARSI score (*p* < 0.01) at 4 weeks compared to the model group (Figure 3). In addition, rats injected with TA alone showed a smooth cartilage surface and a significantly lower OARSI score than that in model group. It is worth noted that the rat joints injected with the hydrogel loaded with TA had the most complete cartilage surface and the lowest OARSI score in all groups.

At 8 weeks, rats in the model group have severe cartilage destruction, with the highest OARSI score (Figure 3). The results of the study showed that the use of TA alone on rat joints can delay the progression of OA. After the injection of the hydrogel loaded with TA, the treatment effect is more remarkable.

### Effect of TA Loaded With GEL-MAN Hydrogel on Joint Morphology and Inflammatory Status of Osteoarthritis in Rats

**FIGURE 4:**
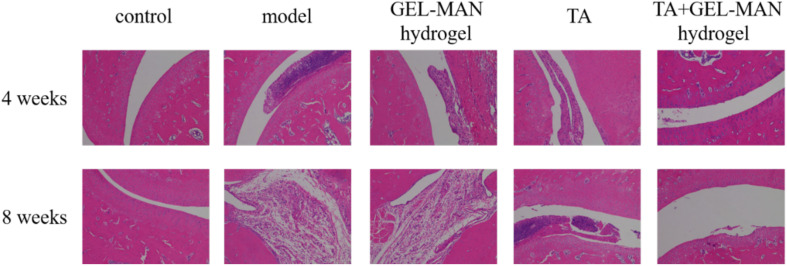
The H&E staining of the rat joint on the 4th and 8th weeks. on the effect of TA loaded with GEL-MAN hydrogel on joint morphology and inflammatory status of osteoarthritis.

We investigated the effect of hydrogel encapsulated TA on the treatment of osteoarthritis in rats by H&E staining (Figure 4). In the control group, the joint structure of the rat was intact at different time points, and no damage and lesions were observed. In the model group, the joint tissue was severely damaged in 4 weeks, and a large number of inflammatory cells, particularly neutrophils, lymphocytes and macrophages infiltrated in the wall of blood vessels. At 8 weeks, the joint structure of the rats was more severely damaged, and some neutrophils, lymphocytes and macrophages accumulated. At 4 weeks, the degree of tissue destruction in the hydrogel group loaded with TA was less than that in the model group. No obvious inflammation was observed. The joint structure was intact, scattered, a small number of fibroblasts were aggregated, and the alveolar septum was not significantly thicker than before. At 8 weeks, fibroblasts increased slightly, and other lesions did not continue to develop. The overall join tissue damage was significantly lighter than the model group.

### Effect of TA Loaded With GEL-MAN Hydrogel on the Degradation of Cartilage Matrix *in vivo*

**FIGURE 5:**
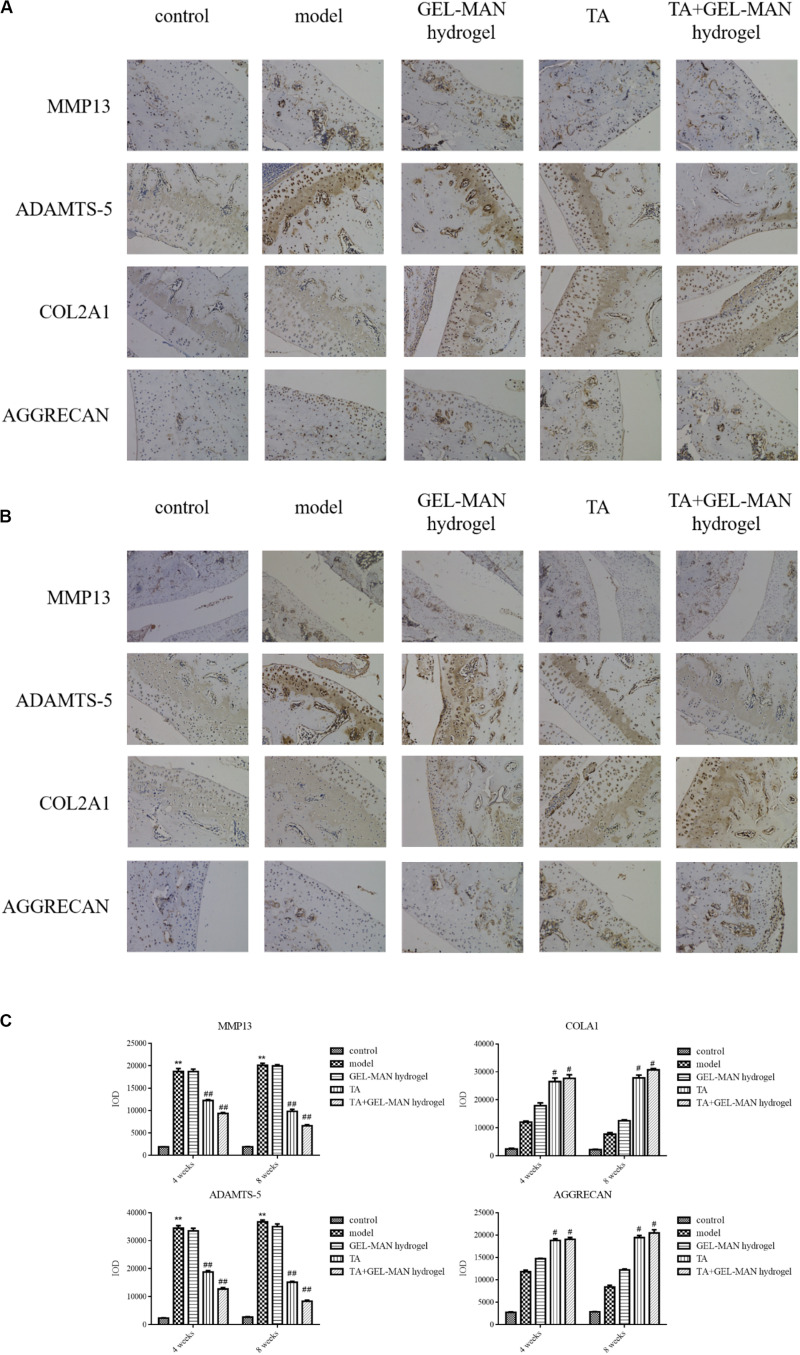
Effect of TA combined with GEL-MAN hydrogel on the degradation of cartilage matrix *in vivo*. **(A,B)** Immunohistochemistry of MMP13, COL2A1, ADAMTS-5, and AGGRECAN at 4 and 8 weeks after OA induction: **(C)** Quantification of MMP13, COL2A1, ADAMTS-5, and AGGRECAN in cartilage samples at 4 and 8 weeks after OA induction. Data are means ± SEM (*n* = 6). # indicates *p* < 0.05 compared with the model group, ## indicates *p* < 0.01 compared with the model group, ** indicates *p* < 0.01 compared with the control group.

In the pathological process of osteoarthritis, the degradation of cartilage matrix often occurs. Therefore, we used immunohistochemistry to examine the expression of biomarkers of cartilage matrix (MMP13, COL2A1, ADAMTS-5, and AGGRECAN) (Figure 5). Rats in the TA + GEL-MAN hydrogel group and the TA-treated group had less MMP13 and ADAMTS-5 expression compared with the model group after the 4th week of treatment (*p* < 0.01). In addition, the expression of MMP13 and ADAMTS-5 were significantly reduced in the TA + GEL-MAN hydrogel group compared with the TA treatment group. In addition, COL2A1 and AGGRECAN-positive chondrocytes had the highest deposition in the TA + GEL-MAN hydrogel group (*p* < 0.05). These results indicated that TA loaded with GEL-MAN hydrogel decreased cartilage matrix degradation.

After 8 weeks, the expression of MMP13 and ADAMTS-5 was observed in the model groups. Notably, the expression of MMP13 and ADAMTS-5 were the lowest in the TA + GEL-MAN hydrogel group (*p* < 0.05). In addition, the expression of COL2A1 and AGGRECAN in the TA + GEL-MAN hydrogel group and the TA-treated group were significantly higher than those in the model group (*p* < 0.05). These results indicated that TA loaded with GEL-MAN hydrogel can effectively prevent cartilage degradation.

## Discussion

The synovial joint has a special anatomical structure, including the synovial membrane, articular cartilage, and joint capsule (Luchetti et al., 2017). Articular cartilage is composed of extracellular matrix and chondrocytes, wherein the content of extracellular matrix accounts for 99%, and chondrocytes only account for about 1%. The extracellular matrix is a fibrous network composed of collagen fibers and proteins. Under normal physiological conditions, the metabolism of the matrix maintains an equilibrium state, so that the articular cartilage has a certain thickness, smooth and elastic, ensuring the normal physiological function of the joint. The pathological manifestation of OA is that the articular cartilage is degraded. First, fine cracks appear on the surface of the cartilage, and then the cartilage layer gradually wears, and finally, the subchondral bone is damaged. In the early stage of cartilage damage, the network structure composed of proteoglycans and collagen fibers is destroyed, resulting in the loss of proteoglycans and ultimately the destruction of the entire structure (Courtney and Doherty, 2009). So far, there is no treatment that can completely cure the disease. The main treatment of OA is to reduce joint pain and restore joint function, often combined with drug therapy and non-drug therapy. Preventive measures include reducing the joint burden by less muscle activity near the joints, changing lifestyle, hyperthermia, acupuncture, and so on.

The main drugs for the treatment of OA are analgesics, non-steroidal anti-inflammatory drugs, glucocorticoids and viscoelastic supplementation (Lau et al., 2015; Zhu et al., 2018). Paracetamol, as a first-line treatment for OA, can effectively alleviate pain in patients. Glucocorticoid is another effective drug for OA treatment, and its therapeutic mechanism on cartilage is diverse. It can reduce synovial inflammation by inhibiting the synthesis of metalloproteinase or synovial pro-inflammatory factor IL-1 (Guidolin, 2018). K. M. Jordan (Zhang et al., 2008). Other studies found that in order to avoid the occurrence of complications, intra-articular injection of glucocorticoids cannot be repeated within 3–6 months. A clinical study of short-term analgesic effects of drugs on 84 patients by Gaffney (Gaffney et al., 1995) found that one of the patients with intra-articular injection of triamcinolone acetonide had to stop midway because the drug leaked from the joint cavity into the systemic circulation. At present, glucocorticoids are used for local injection in the joint cavity. The main side effects include post-injection pain, joint hemorrhage, joint sepsis, steroid cartilage atrophy, systemic toxic reactions, such as fluid retention, increased hypertension, et al. For patients, the compliance of the above methods is generally poor, and surgical treatment is another option for patients. Other OA treatments include joint cavity nutritional supplements, such as injection of glucosamine and chondroitin, can also effectively relieve pain in patients (Zhu et al., 2018).

Compared to systemic administration, topical administration can greatly increase the concentration of drugs at the injection site and reduce systemic side effects. Knee joint injection technology is commonly used for diagnosis and treatment of joint diseases. Due to the small volume of the joint cavity, the actual operation is not easy, since it is difficult for the needle to enter the injection site (Park et al., 2011). In addition, the intra-articular injection may cause infection. Post-injection pain, crystal-induced synovitis, skin atrophy, and steroid joint disease, are the main side effects. Although the incidence of side effects is very low, it will bring great pain to patients once it happens. The intra-articular injection is accompanied by the disadvantages of increased treatment costs, prolonged treatment time and poor patient compliance. Therefore, the latest research on intra-articular injection drug delivery systems must be focus on ensuring the sustained release of the drug at the site of administration while minimizing systemic side effects.

By intra-articular injection, the absorption and distribution of the drug are the same as that of other injections, so a suitable drug delivery system is needed to prolong the residual time of the drug in the joint cavity. Liposomes are an ideal drug carrier, and the current research focuses on increasing the residual time of liposomes at the injection site and improving biocompatibility. Dong et al. (2013) encapsulates dexamethasone palmitate into liposomes, which has a significantly increased residual time compared to microcrystalline triamcinolone acetonide.

In this study, we designed and developed a new material that controls drug release based on the joint condition. The design idea of the hydrogel material is that the material decomposes with the increasing concentration of hydrogen peroxide. Therefore, the hydrogel material loaded with the drug will be injected into the joint cavity, and the hydrogen peroxide will release the drug in the joint synovial fluid of the affected part to treat the diseased joint. Hydrogels are divided into traditional hydrogels that are relatively insensitive to environmental changes and new smart hydrogels that are very sensitive to external conditions. Among them, the new smart hydrogel can respond to different external conditions such as temperature, pH, light source and pressure. The new smart hydrogel is widely used in the field of controlled drug release. The hydrogel acts quickly and accurately and can be directly administered to the site of lesion to increase the local drug concentration and reduce the toxic side effects. The gel undergoes a phase transition at the site of action to form a network structure, and the drug is slowly released by the degradation of the hydrogel network scaffold and its own diffusion, thereby achieving a long-lasting effect.

During the development of osteoarthritis, autophagy acts as a regulatory response to protect cells from various stresses, and failure of autophagy activation can lead to further progression of degeneration (Duarte, 2015). Previous studies on joints in human osteoarthritis and experimental bone and joint models have shown that the reduction in expression of autophagy regulator is associated with increased cell death (Akagi et al., 2017). And related studies have shown that the expression of genes MMP13, COL2A1, ADAMTS-5, and AGGRECAN can be induced by regulating apoptosis and ROS-induced autophagy (Sasaki et al., 2012). MMP13 and ADAMTS-5 are considered to be the major enzymes responsible for the degradation of cartilage matrix. We found that TA can effectively induce autophagy of chondrocytes to reduce MMP13 and ADAMTS-5 and ultimatelyimprove cartilage degeneration. It alleviated the degradation of proteoglycan and collagen, which finally reduce OA syndrome.

In summary, we have developed a new strategy for the treatment of osteoarthritis using GEL-MAN hydrogels for intra-articular release of therapeutic drugs. The combination of TA and GEL-MAN hydrogels can slow the progression of degenerative change in osteoarthritis by maximizing therapeutic efficacy and prolong the duration of treatment.

## Data Availability Statement

All datasets generated for this study are included in the article/supplementary material.

## Ethics Statement

The animal study was reviewed and approved by the Animal Ethics Committee of Tongji University School of Medicine.

## Author Contributions

YZ conceived and designed the study. KC performed the experiments and wrote the first draft. PS assisted designed the hydrogel. SL and YZ edited the manuscript. FY conducted data analyasis. All authors read and approved the final draft.

## Conflict of Interest

The authors declare that the research was conducted in the absence of any commercial or financial relationships that could be construed as a potential conflict of interest.
